# Supplementary material for: Role of Zirconia in Oxide-Zeolite Composite for Thiolation of Methanol with Hydrogen Sulfide to Methanethiol
Source: Nanomaterials (Basel). 2022 May 25;12(11):1803. doi: 10.3390/nano12111803 (PMC9181951; doi:10.3390/nano12111803)
Supplement: Supplementary file 1 [file nanomaterials-12-01803-s001.zip › nanomaterials-1706775-supplementary.pdf]

Supplementary Materials

Role of Zirconia in Oxide-Zeolite Composite for Thiolation of Methanol with Hydrogen Sulfide to Methanethiol

Tinglong Yang [1,2], Mengqin Yao [1,2], Jun Ma [1,2], Peng Chen [1,2], Tianxiang Zhao [1,2], Chunliang Yang [1,2], Fei Liu [1,2,*] and Jianxin Cao [1,2,*]

1 Department of Chemical Engineering, School of Chemistry and Chemical Engineering, Guizhou University, Guiyang 550025, China; tlyangbuaichifan@163.com (T.Y.); mqyao@gzu.edu.cn (M.Y.); jma3@gzu.edu.cn (J.M.); chenpengdeyoujian@163.com (P.C.); guicv2287@foxmail.com (T.Z.); clyang@gzu.edu (C.Y.)

2 Guizhou Key Laboratory for Green Chemical and Clean Energy Technology, Guiyang 550025, China

* Correspondence: ce.feiliu@gzu.edu.cn (F.L.); jxcao@gzu.edu.cn (J.C.)

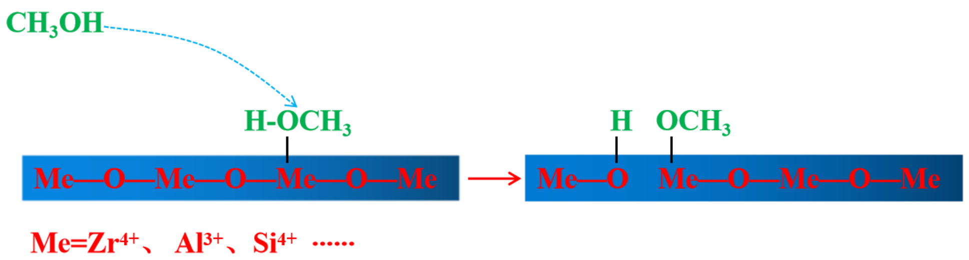

Figure S1. Schematic reaction Mechanism for the formation of methoxy groups.

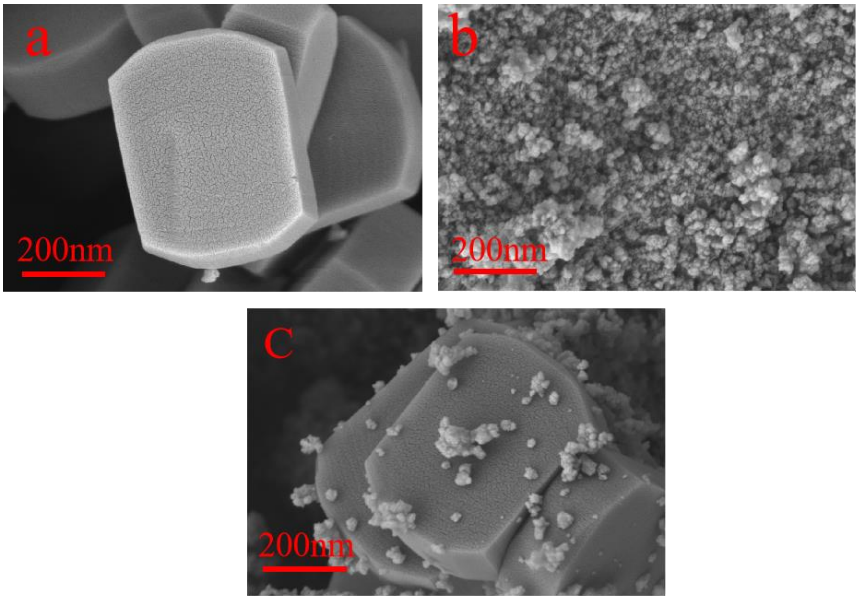

Figure S2. SEM image of the catalyst; (**a**)Pure NaZSM-5, (**b**)Pure m-ZrO_2, (c)m-ZrO_2/NaZSM-5-HC

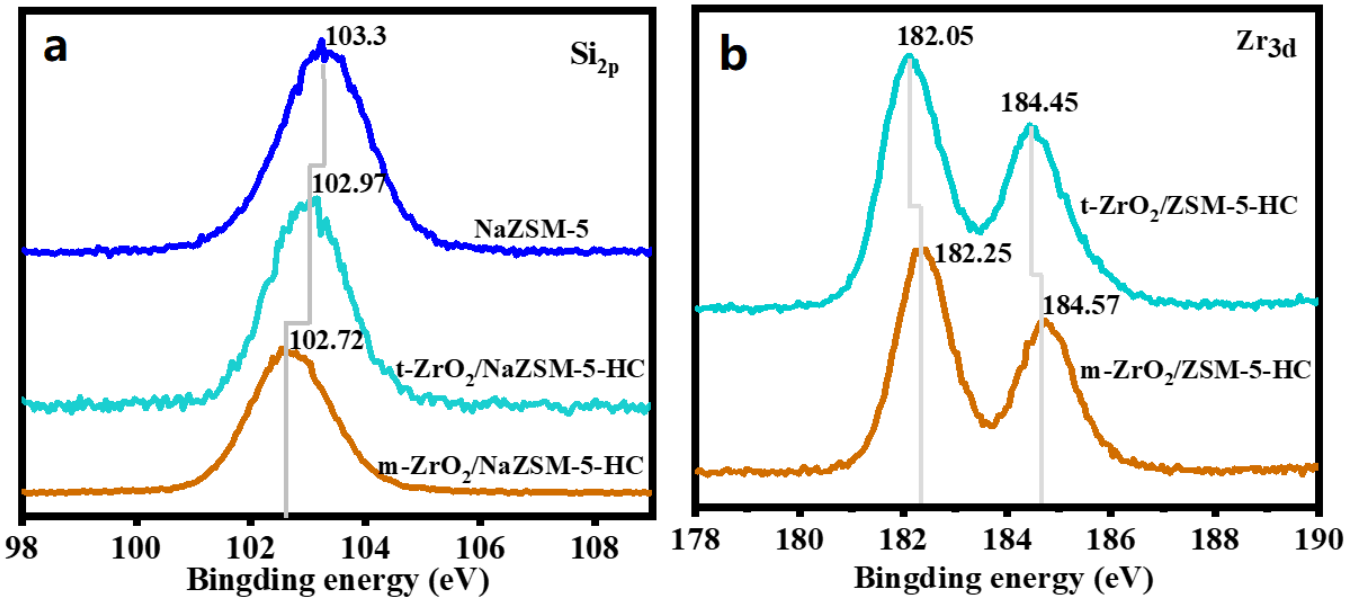

Figure S3. XPS spectra of NaZSM-5, t-ZrO_2/NaZSM-5-HC and m-ZrO_2/NaZSM-5-HC. (**a**)Si_{2p} spectra and (**b**) Zr_{3d} spectra

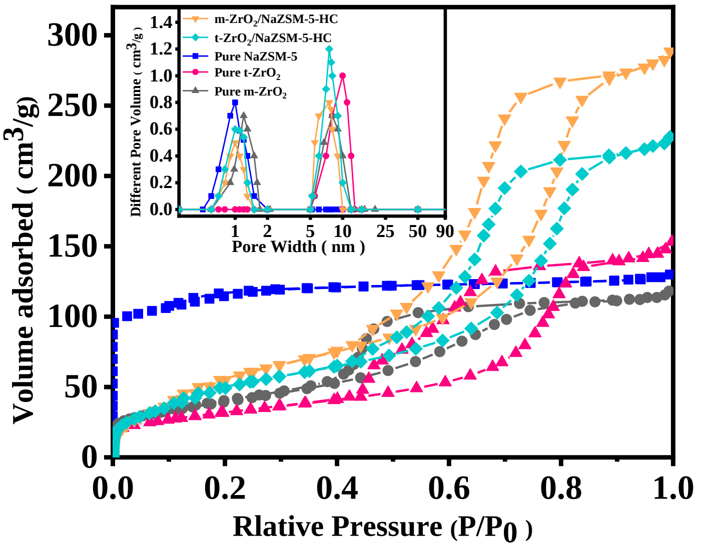

Figure S4. N_2 adsorption-desorption isotherms and pore size distribution of different catalysts

Table S1. The amount of acid and base of different catalysts

Samples	Acid amount (m mol·g^{-1})			Base amount (m mol·g^{-1})		
	Weak acidity (<200°C)	Medium-strong acidity (200~400°C)	Strong acidity (>400°C)	Weak basicity (<200°C)	Medium-strong basicity (200~400°C)	Strong basicity (>400°C)
Pure NaZSM-5	0.081	-	-	0.031	-	-
m-ZrO_2/NaZSM-5-HC	0.035	0.043	-	0.041	0.040	-
t-ZrO_2/NaZSM-5-HC	0.027	0.030	-	0.036	0.041	-
Pure m-ZrO_2	0.017	0.025	-	0.048	-	-
Pure t-ZrO_2	0.021	0.016	-	0.018	0.025	-

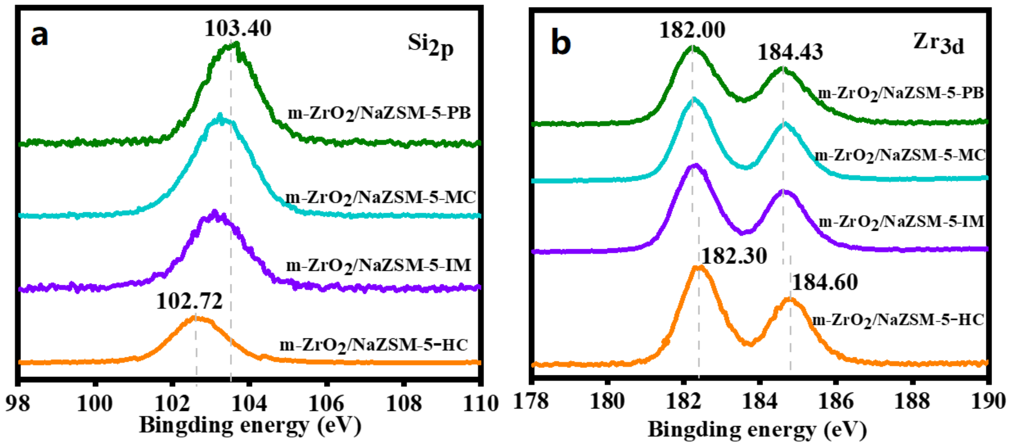

Figure S5. XPS spectra of different composite catalysts. (**a**)Si_{2p} spectra and (**b**) Zr_{3d} spectra

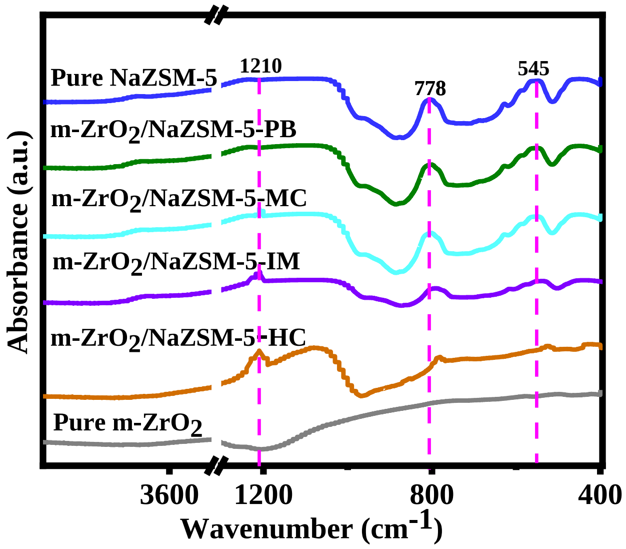

Figure S6. FT-IR spectra of Pure NaZSM-5, Pure m-ZrO_2 and different composite catalysts

Table S2. The amount of acid and base of different catalysts

Samples	Acid amount (m mol·g^{-1})			Base amount (m mol·g^{-1})		
	Weak acidity (<200°C)	Moderate acidit (200~400°C)	Strong acidit (>400°C)	Weak basicity (<200°C)	Moderate basicity (200~400°C)	Strong basicity (>400°C)
m-ZrO_2/NaZSM-5-HC	0.055	0.023	-	0.061	0.020	-
m-ZrO_2/NaZSM-5-IM	0.065	-	-	0.068	-	-
m-ZrO_2/NaZSM-5-MC	0.063	-	-	0.060	-	-
m-ZrO_2/NaZSM-5-PB	0.056	-	-	0.039	-	-

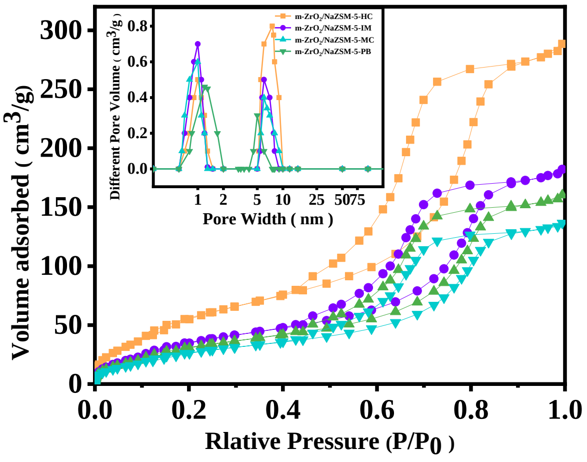

Figure S7. N_2 adsorption-desorption isotherms and pore size distribution of different composite catalysts
